# Association between Carotid Intima-Media Thickness and Cognitive Impairment in a Chinese Stroke Population: A Cross-sectional Study

**DOI:** 10.1038/srep19556

**Published:** 2016-01-25

**Authors:** Wei Yue, Anxin Wang, Hui Liang, Fengyun Hu, Yunshu Zhang, Meiying Deng, Tong Li, Xiao Hu, Zhenxuan Ye, Ying Shen, Yong Ji

**Affiliations:** 1 Department of Neurology, Tianjin Huanhu Hospital, Tianjin, China; 2 Department of Neurology, Beijing Tiantan Hospital, Capital Medical University, Beijing, China; 3 Department of Epidemiology and Health Statistics, School of Public Health, Capital Medical University, Beijing, China; 4 Department of Neurology, The First Affiliated Hospital, Zhejiang University, Zhejiang, China; 5 Department of Neurology, Shanxi People’s Hospital, Shanxi Medical University, Shanxi, China; 6 Department of Neurology, The Third Hospital of Shijiazhuang, Shijiazhuang, China; 7 Department of Neurology, The First Affiliated Hospital of Xi’an Jiao Tong University, Xian, China; 8 Department of Neurology, The First Affiliated Hospital of Xinxiang Medical College, Xinxiang, China; 9 Department of Neurology, Guizhou People’s Hospital, Guizhou, China; 10 Department of Inspection, Guizhou People’s Hospital, Guizhou, China; 11 Department of Traditional Chinese Medicine, Beijing Xuanwu Hospital, Capital Medical University, Beijing, China

## Abstract

This study aimed to investigate the potential associations between carotid intima-media thickness and cognitive impairment among patients with acute ischemic stroke and to identify the clinical implications. We measured carotid intima-media thickness (IMT) and performed the Mini-Mental State Examination (MMSE) upon the admission of 1,826 acute ischemic stroke patients. The association between IMT and cognitive impairment evaluated by the MMSE was assessed with a multivariate regression analysis. Other clinical variables of interest were also assessed. After adjusting for potential confounders, participants in the highest IMT quartile had a higher likelihood of having cognitive impairments compared with the lowest IMT quartile (odds ratio: 3.01, 95% confidence interval: 2.07–4.37, p < 0.001). Stratified analyses indicated that this positive correlation was similar for the maxIMT and meanIMT of carotid artery measurements. A positive correlation was found between IMT and cognitive impairment in participants with acute ischemic stroke.

Cognitive impairment is associated with disability and care dependence worldwide. Cognitive impairment may be the earliest, most common, and subtlest manifestation of cerebrovascular disease^1^,^2^. Previous studies have suggested that silent stroke may occur concurrently with vascular risk factors^3^ and cause accumulated cognitive decline^4^. Western studies have investigated the association between vascular risk factors and cognitive impairment in elderly individuals^5^,^6^,^7^,^8^ and demonstrated that carotid atherosclerosis is a risk factor for cognitive impairment. Few studies have investigated the relationship between carotid atherosclerosis and cognitive impairment in younger adults^9^,^10^. Previous epidemiological studies have indicated an association between carotid atherosclerosis and cognitive decline in stroke-free individuals, but the results from population-based studies have been less consistent. Hachinski *et al*. reported that one sixth of all patients exhibit cognitive impairment prior to an acute stroke, and one third of all patients develop impairment after an acute stroke^1^. In addition, cerebral infarction contributes to cognitive impairment in approximately 50% of cases^11^ and is occasionally associated with Alzheimer’s disease^12^. Until now, limited studies have investigated the associations between carotid intima-media thickness (IMT) and cognitive impairment in stroke patients^13^,^14^.

We therefore conducted this study to examine the relationship between IMT, stratified by max and mean value, and cognitive function in stroke individuals.

## Results

In total, 1,184 men and 642 women were enrolled in this study (mean age, 63.20 ± 11.92 years). Ultrasound images and neuropsychological data were obtained. Among the study population, 513 (28.09%) patients were diagnosed with cognitive impairment, which was defined as an MMSE score <24. The mean maxIMT was 1.37 ± 0.71 mm.

Table 1 presents the characteristics of patients with good cognition and cognitive impairment. Age, sex, education, alcohol use, tobacco use, history of hypercholesterolemia, and atrial fibrillation were associated with cognitive impairment (p < 0.05). Compared to patients with good cognition, patients with cognitive impairment had a higher median NIHSS score (6 vs. 3, p < 0.05) and an increased maxIMT (1.54 vs. 1.30, p < 0.05) and meanIMT (1.10  vs. 1.05, p < 0.05). Likewise, compared to patients with good cognition, patients with cognitive impairment had a higher proportion of large artery stroke, cardioembolism, and other reasons stroke and a lower proportion of lacunar infarctions (p < 0.05).

The relationships between the quartiles of maxIMT and background characteristics are summarized in Table 2. No differences in sex, alcohol use, tobacco use, education level, history of hypertension, diabetes, hypercholesterolemia, atrial fibrillation, or coronary artery disease were found; however, age, marital status, physical activity, NIHSS score, and stroke subtypes were significantly different between varying IMT quartiles (p < 0.05).

A multivariate regression analysis was performed to investigate the associations between cognitive function and maxIMT or meanIMT (Table 3). After adjusting for potential confounders, participants in the highest IMT quartile had a greater likelihood of having cognitive impairment compared with those in the lowest IMT quartile (odds ratio (OR): 3.01, 95% confidence interval (CI): 2.07–4.37, p < 0.001). Every 0.1 mm increase in maxIMT was associated with a mean 4% increase in cognitive impairment. In addition, the association between meanIMT and cognitive impairment was also investigated. Compared to participants in the lowest IMT quartile, participants in the highest IMT quartile had a greater likelihood of having cognitive impairment (OR: 2.13, 95% CI: 1.48–3.08, p < 0.001) (Table 3).

## Discussion

Our study explored the effect of carotid intima-media thickness on cognitive function in a stroke population. We observed a positive association between IMT and cognitive impairment in acute ischemic stroke patients.

In the present study, increased IMT was correlated with cognitive impairment in stroke patients older than 18 years, which is consistent with previous reports on non-stroke patients^6^,^8^,^9^,^15^,^16^,^17^. This association was independent of known confounding factors, such as age, sex, blood parameters, education level, marital status, alcohol use, tobacco use, physical activity, history of hypertension, diabetes, hypercholesterolemia, atrial fibrillation, coronary artery disease, stroke subtypes, and NIHSS score. Our data support the previously published results of the Rotterdam study^18^. This study observed a strong association between IMT and risk of cognitive impairment. In accord with our results, another large prospective study found that subjects with greater internal carotid artery IMT had an increased risk of developing Alzheimer’s disease^19^. In a prospective study of 10,963 subjects aged 47–70 years, no association was found between mean carotid IMT and cognitive decline after a 6-year follow-up^16^. A recent study of 1,130 subjects (59 years old at baseline) with a 14-year follow-up found no association between carotid IMT and cognitive decline^20^.

Because plaque thickness was included in our measurements, we investigated the associations between maxIMT and cognitive impairment and between meanIMT and cognitive impairment. After adjusting for risk factors of vascular disease, the associations remained significant for both the maxIMT and meanIMT of common carotid arteries. These findings are consistent with the hypothesis that IMT is a marker for underlying risk factors and generalized atherosclerosis rather than a direct cause of cognitive impairment. However, these associations were of smaller magnitude and not significant in a study by Johnston^8^. Two possible explanations may have caused this discrepancy. First, our population included patients diagnosed with acute ischemic stroke. Second, differences in the ethnicities of the participants may contribute to these differences. Our participants were primarily Han ethnic Chinese, who likely have different physiques and lifestyles compared with other populations.

Possible pathophysiological explanations for the relationship between increased IMT and cognitive impairment should be considered. Arterial wall thickening may cause the narrowing of the vessel lumen, decreased intracranial arterial perfusion pressure, and reduced blood flow velocity (leading to hypoperfusion). These effects may result in chronic ischemia and reduced energy supply, eventually leading to cognitive dysfunction^21^. In addition, measures of carotid atherosclerosis are associated with various cardiovascular risk factors, including demographic, metabolic, immunologic, and lifestyle factors, which are associated with poorer cognitive function^15^.

An important strength of our study was the evaluation of various covariates for cognitive impairment, such as education level, physical activity, atrial fibrillation, coronary artery disease, stroke subtypes, and NIHSS, which were not included in the Rotterdam study. In addition, this study had a multi-center design and was based on a randomly selected population of acute ischemic stroke patients in 43 hospitals across China.

Although the sample size was large and we adjusted for a variety of potential confounders, several limitations should be noted. First, the sample consisted of mostly Chinese Han individuals, and the mean education level was high compared with the general Chinese population. Therefore, our results may not be generalizable to the population of China. Second, cognitive impairment in our study was only assessed by the MMSE, although vascular cognitive decline disproportionately affects executive function^22^. Finally, we did not collect the information on the site of the stroke event, which could bias the results. Strokes affecting different cortical areas can lead to a different probability of impairment.

## Methods

### Study Design and Population

The present cohort was obtained from the Study on Oxidative Stress in Patients with Acute Ischemic Stroke (SOS-Stroke), a prospective, multi-center registry. The SOS-Stroke study consisted of consecutively selected patients (n = 4,164) with acute ischemic stroke. Patients (age range 18 to 90 years) who had suffered a stroke and were admitted to one of the 43 designated hospitals in China within 7 days were included in this study from January to October 2014. The inclusion criteria for SOS-stroke were as follows: (1) over 18 years of age; (2) neurologist diagnosed the patient with acute ischemic stroke that was confirmed with computed tomography (CT) or magnetic resonance imaging (MRI); (3) time from initial stroke to diagnosis was less than two weeks; and (4) patient provided informed consent. The exclusion criteria were as follows: (1) bleeding or other pathological brain diseases, such as vascular malformations, tumors, abscesses, multiple sclerosis, or other common non-ischemic cerebral disease, revealed via head CT and/or MRI; (2) transient ischemic attack (TIA); and (3) iatrogenic stroke due to angioplasty or surgical operations. We excluded 417 participants with incomplete MMSE data and 1,921 participants with incomplete IMT data. Finally, only 1,826 participants (1,184 men, 642 women) were kept for data analysis. The study was sponsored by the China Stroke Prevention Project of the National Health and Family Planning Commission and was approved by the local ethics committees in compliance with the Declaration of Helsinki. All patients provided informed consent prior to participation.

### Biometric Indicators

Blood pressure was measured with a mercury sphygmomanometer with an appropriately sized cuff. Two readings (five-minute interval) of systolic blood pressure (SBP) and diastolic blood pressure (DBP) were obtained after the participants had rested in a chair for at least five minutes. The mean of the two readings was used for the analyses. If the two measurements differed by more than 5 mmHg, an additional reading was obtained, and the mean of the three readings was used.

Blood samples were drawn by trained phlebotomists after overnight fasting. Serum levels of fasting blood glucose (FBG), total cholesterol (TC), high-density lipoprotein (HDL) cholesterol, and low-density lipoprotein (LDL) cholesterol were assessed. All venous blood samples were obtained in the morning following the fasting period, and the serum was centrifuged and frozen within 48 hours at −15 °C to −20 °C.

### Assessment of Potential Covariates

Information regarding demographic and clinical characteristics (age, sex, marital status, alcohol use, education, and history of diseases) was collected via questionnaires. Marital status was stratified as married or unmarried (including single, divorced, or widowed). Alcohol use was defined as a daily intake of at least 100 ml of liquor three times per week for more than one year. Physical activity was evaluated regarding the type and frequency of physical activity at work and during leisure time. Previous history of disease, including myocardial infarction, stroke, hypertension, diabetes, hypercholesterolemia, atrial fibrillation, and coronary artery disease, was determined via self-reporting. The use of antihypertensive, cholesterol-lowering, and glucose-lowering medications within the two weeks prior to the baseline interview was also self-reported.

### Ultrasound Examination

Carotid intima-media thickness (IMT) was measured by local experienced investigators with high-resolution B-mode ultrasonography with a 7.5-MHZ probe based on a slight modification of the Atherosclerosis Risk in Communities (ARIC) protocol^23^,^24^. Each participant had 10 or more images collected from the near and far walls of the right and left common carotid arteries (CCAs). If plaque was present in the common carotid artery segments, the IMT score included these measurements. Therefore, the IMT of the CCAs was defined as the mean (meanIMT) and maximum (maxIMT) of the maximum intima-media thicknesses of the near and far walls, respectively.

### Neuropsychological Evaluation

Cognitive function was measured annually using the Mini-Mental State Examination (MMSE). The MMSE is a measure of general cognitive function and includes orientation to time and place, attention and calculation, language, and memory^25^. Higher scores indicate greater cognitive function. Cognitive impairment was defined as a score of less than 24.

### Statistical Analyses

Statistical analyses were performed using a commercially available software program (SAS software, version 9.4; SAS Institute Inc., Cary, NC, USA). Data are presented as the means ± SD for continuous variables and as frequencies and percentages for categorical variables. We used Student’s t-test or ANOVA for non-paired samples to compare normally distributed parameters and the Wilcoxon or Kruskal-Wallis tests to compare non-parametric variables. The Chi-squared test was applied to compare categorical variables. Third, the entire study population was divided into four groups according to IMT quartile: quartile 1 (≤0.89 mm), quartile 2 (0.90 ≤ IMT ≤ 1.15 mm), quartile 3 (1.20 ≤ IMT ≤ 1.40 mm), and quartile 4 (IMT ≥ 1.50). Variables were compared between these four subgroups. Lastly, multivariate odds ratios (OR) were obtained via logistic regression analysis after adjusting for possible confounders, including age, sex, blood parameters, education level, marital status, alcohol use, tobacco use, physical activity, history of hypertension, diabetes, hypercholesterolemia, atrial fibrillation, coronary artery disease, stroke subtypes, and National Institutes of Health Stroke Scale (NIHSS) score. A p-value less than 0.05 (2-sided) was considered significant.

## Additional Information

**How to cite this article**: Yue, W. *et al*. Association between Carotid Intima-Media Thickness and Cognitive Impairment in a Chinese Stroke Population: A Cross-sectional Study. *Sci. Rep.* **6**, 19556; doi: 10.1038/srep19556 (2016).

## Figures and Tables

**Table 1:** Comparisons between patients with and without cognitive impairment among a population with stroke.

**Variable**	Cognitively intact(MMSE **≥** **24) n** **=** **1313**	Cognitively impaired(MMSE **<** **4) n** **=** **513**	**p value**
61.81 ± 11.66	66.77 ± 11.84	<0.0001	
Male, n (%)	904 (68.85%)	280 (54.58%)	<0.0001
Married, n (%)	1,246 (94.90%)	474 (92.40%)	0.0401
SBP, mmHg	147.18 ± 22.38	148.96 ± 22.68	0.0843
DBP, mmHg	85.51 ± 12.50	84.02 ± 13.63	0.0084
FBG, mmol/l	6.57 ± 2.79	6.57 ± 2.86	0.8276
TC, mmol/l	4.66 ± 1.31	4.63 ± 1.63	0.3309
HDL, mmol/l	1.14 ± 0.35	1.16 ± 0.42	0.4455
LDL, mmol/l	2.91 ± 1.32	2.86 ± 1.21	0.3786
Alcohol use (Yes)	453 (34.50%)	127 (24.76%)	<0.0001
Tobacco use (Yes)	554 (42.19%)	155 (30.21%)	<0.0001
Physical activity (Yes)	480 (36.56%)	184 (35.87%)	0.7829
Education level, n (%)			
Illiteracy/primary	1,181 (89.95%)	453 (88.30%)	0.3037
Middle school and above	132 (10.05%)	60 (11.70%)	
History of diseases, n (%)			
Hypertension	876 (66.72%)	347 (67.64%)	0.7060
Diabetes	314 (23.91%)	104 (20.27%)	0.0959
Hypercholesterolemia	166 (12.64%)	41 (7.99%)	0.0048
Atrial fibrillation	47 (5.58%)	32 (6.24%)	0.0121
Coronary artery disease	29 (2.21%)	14 (2.73%)	0.5098
Stroke subtypes, n (%)			
Large artery stroke	694 (52.86)	322 (62.77)	<0.0001
Cardioembolism	50 (3.81)	38 (7.41)	
Lacunar infarctions	502 (38.23)	103 (20.08)	
Other reasons	16 (1.22)	24 (4.68)	
Unknown reason	51 (3.88)	26 (5.07)	
NIHSS, [median (IQR)]	3 (2–6)	6 (4–11)	<0.0001
MaxIMT, mm	1.30 ± 0.68	1.54 ± 0.74	<0.0001
MeanIMT, mm	1.05 ± 0.47	1.10 ± 0.40	<0.0001

SBP: systolic blood pressure; DBP: diastolic blood pressure; FBG: fasting blood glucose; TC: total cholesterol; HDL: high-density lipoprotein cholesterol; LDL: low-density lipoprotein cholesterol.

Common IMT: intima-media thickness of the common carotid artery; MMSE: mini-mental state examination; SD: standard deviation; NIHSS: National Institutes of Health Stroke Scale.

**Table 2:** Demographic and Clinical Characteristics Stratified by Quartiles of maximum Carotid Intima-media Thickness (maxIMT).

**Characteristic**	**1**^**st**^ **Quartile**	**2**^**nd**^ **Quartile**	**3**^**rd**^ **Quartile**	**4**^**th**^ **Quartile**	**p value**
Age, years	61.22 ± 12.15	63.71 ± 12.13	63.53 ± 11.79	64.10 ± 11.43	0.0029
male, n (%)	254 (61.65%)	326 (65.07%)	293 (65.70%)	311 (66.60%)	0.4532
Married (Yes)	403 (97.82%)	467 (93.21%)	408 (91.48%)	442 (94.65%)	0.0007
SBP, mmHg	148.97 ± 23.72	145.60 ± 21.66	149.47 ± 22.07	147.05 ± 22.44	0.0206
DBP, mmHg	85.49 ± 12.65	83.89 ± 12.41	85.39 ± 12.53	85.76 ± 13.67	0.0213
FBG, mmol/l	6.74 ± 3.05	6.33 ± 2.51	6.70 ± 2.95	6.55 ± 2.74	0.1292
TC, mmol/l	4.57 ± 1.34	4.50 ± 1.32	4.82 ± 1.73	4.72 ± 1.19	0.0281
HDL, mmol/l	1.08 ± 0.30	1.17 ± 0.42	1.14 ± 0.32	1.21 ± 0.39	0.0001
LDL, mmol/l	2.78 ± 1.03	2.83 ± 1.71	2.96 ± 1.18	2.99 ± 1.05	0.0006
Alcohol use (Yes)	136 (33.01%)	155 (30.94%)	132 (29.60%)	157 (33.62%)	0.5397
Tobacco use (Yes)	163 (39.56%)	189 (37.72%)	170 (38.12%)	187 (40.04%)	0.8648
Physical activity (Yes)	136 (33.01%)	190 (37.92%)	138 (30.94%)	200 (42.83%)	0.0008
Education level, n (%)					
Illiteracy/primary	368 (89.32%)	454 (90.62%)	405 (90.81%)	407 (87.15%)	0.2380
Middle school or above	44 (10.68%)	47 (9.38%)	41 (9.19%)	60 (12.85%)	
History of diseases, n (%)					
Hypertension	262 (63.59%)	351 (70.06%)	309 (69.28%)	301 (64.45%)	0.0820
Diabetes	99 (24.03%)	106 (21.16%)	111 (24.89%)	102 (21.84%)	0.4837
Hypercholesterolemia	40 (9.71%)	48 (9.58%)	60 (13.45%)	59 (12.63%)	0.1452
Atrial fibrillation	21 (5.10%)	23 (4.59%)	11 (2.47%)	24 (5.14%)	0.1612
Coronary artery disease	5 (1.21%)	13 (2.59%)	13 (2.91%)	12 (2.57%)	0.3675
Stroke subtypes, n (%)					
Cardioembolism	25 (6.07)	27 (5.39)	14 (3.14)	22 (4.71)	0.0126
Large artery stroke	235 (57.04)	267 (53.29)	241 (54.04)	273 (58.46)	
Lacunar infarctions	127 (30.83)	177 (35.33)	169 (37.89)	132 (28.27)	
Other reasons	6 (1.46)	7 (1.40)	8 (1.79)	19 (4.07)	
Unknown reason	19 (4.61)	23 (4.59)	14 (3.14)	21 (4.50)	
NIHSS, [median (IQR)]	3 (2–7)	4 (2–6)	4 (2–7)	5 (2–7)	0.0001
MMSE score	26.23 ± 6.02	24.62 ± 7.12	24.21 ± 6.92	23.04 ± 8.19	<0.0001
MaxIMT, mm	0.70 ± 0.14	1.01 ± 0.07	1.28 ± 0.08	2.43 ± 0.51	<0.0001
MeanIMT, mm	0.65 ± 0.13	0.96 ± 0.11	1.18 ± 0.12	1.43 ± 0.66	0.0001

SBP: systolic blood pressure; DBP: diastolic blood pressure; FBG: fasting blood glucose; TC: total cholesterol; HDL: high-density lipoprotein cholesterol; LDL: low-density lipoprotein cholesterol.

Common IMT: intima-media thickness of the common carotid artery; MMSE: mini-mental state examination; NIHSS: National Institutes of Health Stroke Scale.

**Table 3:** Odds Ratios of Cognitive Impairment Stratified by maximum and mean Common Carotid Artery Intima-media Thickness Quartiles.

	**1**^**st**^ **Quartile**	**2**^**nd**^ **Quartile**	**3**^**rd**^ **Quartile**	**4**^**th**^ **Quartile**	**1** **mm increase in IMT**	**p for trend**
MaxIMT						
Model 1*	1.00	1.78 (1.28–2.48)	2.00 (1.43–2.80)	3.01 (2.17–4.18)	1.05 (1.03–1.06)	<0.0001
Model 2†	1.00	1.94 (1.38–2.72)	2.17 (1.53–3.06)	3.32 (2.37–4.65)	1.05 (1.03–1.06)	<0.0001
Model 3‡	1.00	2.03 (1.40–2.96)	2.18 (1.49–3.18)	3.01 (2.07–4.37)	1.04 (1.02–1.06)	<0.0001
Mean IMT						
Model 1*	1.00	1.66 (1.22–2.26)	2.11 (1.55–2.89)	1.72 (1.26–2.36)	1.02 (1.00–1.04)	<0.0001
Model 2†	1.00	1.17 (1.25–2.37)	2.23 (1.62–3.08)	1.96 (1.41–2.72)	1.03 (1.01–1.06)	<0.0001
Model 3‡	1.00	1.99 (1.40–2.84)	2.34 (1.63–3.34)	2.13 (1.48–3.08)	1.03 (1.00–1.05)	<0.0001

^*^Adjusted for age (years), sex.

^†^Adjusted as model 1 plus education level (elementary school, high school or college or above), marital status, systolic blood pressure, diastolic blood pressure, fasting blood glucose, total cholesterol, high-density lipoprotein cholesterol, low-density lipoprotein cholesterol, alcohol use, tobacco use, physical activity, history of hypertension, diabetes, hypercholesterolemia, atrial fibrillation, and coronary artery disease.

^‡^Adjusted as model 2 plus stroke subtypes and NIHSS score.
